# Capicua Regulates Dendritic Morphogenesis Through Ets in Hippocampal Neurons *in vitro*

**DOI:** 10.3389/fnana.2021.669310

**Published:** 2021-07-27

**Authors:** Keqin Li, Shuai Shao, Tongjie Ji, Min Liu, Lufeng Wang, Ying Pang, Mu Chen, Siyi Xu, Kuiming Zhang, Qi Wang, Zhongwei Zhuang, Liang Wei, Yanfei Zhang, Yanlin Chen, Yang Wang, Jing Zhang, Kui Chen, Hao Lian, Chunlong Zhong

**Affiliations:** ^1^Department of Neurosurgery, Shanghai East Hospital, Tongji University School of Medicine, Shanghai, China; ^2^Department of Neurosurgery, Ren Ji Hospital, School of Medicine, Shanghai Jiao Tong University, Shanghai, China; ^3^Department of Neurology, Shanghai East Hospital, Tongji University School of Medicine, Shanghai, China; ^4^Department of Emergency, Xinhua Hospital Affiliated to Shanghai Jiao Tong University School of Medicine, Shanghai, China

**Keywords:** capicua, transcriptional repressor, dendritic growth, spine growth, Ets factors

## Abstract

Capicua (Cic), a transcriptional repressor frequently mutated in brain cancer oligodendroglioma, is highly expressed in adult neurons. However, its function in the dendritic growth of neurons in the hippocampus remains poorly understood. Here, we confirmed that Cic was expressed in hippocampal neurons during the main period of dendritogenesis, suggesting that Cic has a function in dendrite growth. Loss-of-function and gain-of function assays indicated that Cic plays a central role in the inhibition of dendritic morphogenesis and dendritic spines *in vitro*. Further studies showed that overexpression of Cic reduced the expression of Ets in HT22 cells, while *in vitro* knockdown of Cic in hippocampal neurons significantly elevated the expression of Ets. These results suggest that Cic may negatively control dendrite growth through Ets, which was confirmed by ShRNA knockdown of either Etv4 or Etv5 abolishing the phenotype of Cic knockdown in cultured neurons. Taken together, our results suggest that Cic inhibits dendritic morphogenesis and the growth of dendritic spines through Ets.

## Introduction

Dendritic arborization and spine formation are critical for the function of neurons. Growing evidence has established that many neurodevelopmental disorders, such as schizophrenia (Lima Caldeira et al., 2019), bipolar disorder (Konopaske et al., 2014), autism spectrum disorder (Bagni and Zukin, 2019), and intellectual disability (Quach et al., 2020), are caused by structural abnormalities of dendrites and their connections. Dendritic spine damage is also a feature of Alzheimer’s disease (Liu et al., 2013), Parkinson’s disease (Nishijima et al., 2018) and traumatic brain injury (Xiong et al., 2019). Thus, it is necessary to clarify the mechanisms of dendritic arborization and abnormalities in the pathogenesis of neurological disorders.

The proper formation and morphogenesis of dendrites are highly controlled by both external signals and intrinsic genetic programs (Ledda and Paratcha, 2017). Extrinsic factors include secreted neurotrophic factors, cell adhesion molecules, and activity-dependent calcium signals, which need to be effectively translated to changes in transcription, translation, cytoskeleton dynamics, and membrane trafficking (Ledda and Paratcha, 2017). The intrinsic cues regulating dendrite morphogenesis include transcription factors, cytoskeletal regulators and motor proteins as well as secretory membrane pathways and regulatory RNAs (de la Torre-Ubieta and Bonni, 2011; Ledda and Paratcha, 2017). Transcription factors, which are some of the most important inherent genetic regulators, control the entire developmental program of neurons and alter the response to extrinsic signals (de la Torre-Ubieta and Bonni, 2011).

Capicua (Cic), a member of the highly conserved high mobility group (HMG) box superfamily of transcription factors, promotes tumor progression and metastasis by directly controlling the transcription of effector target genes across human cancers (Lee, 2020; Kim et al., 2021). Beyond its clear role in cancer, the function of Cic in hippocampal neurons has not been well studied. Here, we found that Cic was highly expressed in the hippocampus of mice. Loss- and gain-of-function assays were used to determine its role in dendritic outgrowth in the hippocampus. Our results revealed that Cic negatively regulates dendritic arborization and spine formation. Furthermore, we found that these effects were possibly mediated through Etv4 and Etv5.

## Materials and Methods

### Plasmid Construction and Lentivirus Packaging

The Cic DNA constructs (pcDNA3.1(+)-Cic-3xFLAG-P2A-EGFP) were provided by OBiO Technology Corporation (Shanghai, China). Lentiviral plasmids were designed against mouse Cic or a negative control, and lentiviruses were obtained from Genomeditech (Shanghai, China). The Cic shRNA sequences were as follows: ShRNA1: GCAGGTGCCAGGACTGAAATG; ShRNA2: GCATC ATACTCCGGCCCAAAG; ShRNA3: GCGGGAGAAGGACCAT ATTCG; and scramble shRNA (ShSCR): TTCTCCGAA CGTGTCACGT. All ShRNAs mentioned above were constructed in the pGMLV-SC5-EGFP vector. ShRNA1-Cic and ShSCR were also constructed in the pSLenti-CMV-WPRE vector. The Etv4 shRNA sequences were as follows: ShRNA1: GGATGAAAGGCGGATACTT; ShRNA2: GCAGCAAATCTCCCGGAAA; and ShRNA3: GGACCTCAGTCACTTCCAA. The Etv5 shRNA sequences were as follows: ShRNA1: GCAGGAATACCATGACCCA; ShRNA2: GCCAGTCATCCTACATGAG; and ShRNA3: GCTCTCTCCGCTATTACTA. All Etv4 ShRNAs and Etv5 ShRNAs were constructed in the pSLenti-CMV-WPRE vector.

### HT22 Cell Culture and Transfection

The hippocampal cell line HT22 was acquired from the Cell Bank of Shanghai Institute of Cells, Chinese Academy of Sciences (Shanghai, China). Cells were incubated in Dulbecco’s modified Eagle medium (DMEM) supplemented with 10% fetal bovine serum (FBS, Gibco, Grand Island, NY, United States) and 1% penicillin/streptomycin sulfate (PB180120, Procell Life Science and Technology Co., Ltd.). The cells were cultured at 37°C in a humidified 5% CO_2_ incubator. Lipofectamine^TM^ 3000 transfection reagent (Invitrogen/Thermo Fisher Scientific) was used for plasmid transfection following the manufacturer’s protocol.

### Primary Neuron Cell Cultures and Transfection

To obtain primary hippocampal neurons from mice, embryonic day 18 (E18) mouse brains were obtained as previously described (Sun et al., 2014). Dissociated neurons were seeded onto poly-D-lysine and cultured in a neurobasal medium (Invitrogen, Carlsbad, CA) supplemented with B27 (Invitrogen), 0.5 mM glutamine, 12.5 μM glutamate, and penicillin/streptomycin. The calcium phosphate method was used to transfect hippocampal neurons with the indicated plasmids for 6–14 days at 7 days *in vitro* (DIV). In the case of transfection with two plasmids, pGMLV-SC5-EGFP was mixed with two other plasmids at a ratio of 1:2:2.

### Western Blotting

Cells were harvested and lysed at 4°C in RIPA lysis buffer (Beyotime, P0013) supplemented with 1 mM PMSF and protease inhibitor cocktail (Thermo, 78,425). Total protein lysates were separated in 7.5 or 10% sodium dodecyl sulfate-polyacrylamide gels (Epizyme, PG111, and PG112, respectively) and transferred to polyvinylidene fluoride membranes (Millipore). The primary antibodies were diluted in blocking buffer, and the membranes were incubated with the primary antibodies at 4°C overnight and then with HRP-conjugated anti-rabbit and anti-mouse secondary antibodies (Beyotime, A0208, and A0216, respectively). A chemiluminescence detection system (Tianneng, China) was used to visualize the protein bands. Quantification was performed by analyzing the relative density of the immunoreactive bands using ImageJ. For Wb the primary antibodies we used rabbit anti-Cic (1:1,000, Thermo Fisher Scientific, PA1-46018), mouse anti-GAPDH (1:5,000, Sigma-Aldrich, G8795), rabbit anti-α-Tubulin (1:2,000, Cell Signaling Technology, 2148s), rabbit anti-Lamin B1 (1:1,000, Beyotime, AF1408), Etv4 (1:500, Santa Cruz, sc-166629), and Etv5 (1:500, Santa Cruz, sc-100941).

### Immunofluorescence (IF)

Primary cultured neurons were fixed in PBS containing 4% PFA for 30 min at 4°C. For brain slice immunofluorescence assays, mice were transcardially perfused with 4% PFA, the brains were fixed with 4% PFA at 4°C overnight, and 30-μm coronal brain slices were obtained. The cells or brain slices were then blocked with blocking buffer (0.3% Triton X-100 in PBS) containing 10% donkey serum (Jackson ImmunoResearch, 122,346) for 1 h at room temperature. Then, the cells or slices were incubated with primary antibodies in blocking buffer containing 2% goat serum overnight at 4°C. The following primary antibodies were used for dual immunofluorescence: rabbit anti-Cic (1:1000 for cells, 1:200 for slices, Thermo Fisher Scientific, PA1-46018) and chicken anti-Map2 (1:10,000 for cells, 1:1,000 for slices, Aves Labs, MAP). Cell nuclei were stained with DAPI.

### Image Analysis and Quantification

For the analysis of dendritic morphology, cell images were obtained with a confocal microscope (Leica SP8). Objectives of 20, 40, and 63× were used. Morphometric analysis and quantification were performed as recently described (Chen et al., 2020). Briefly, a z-series of 6–12 images with a 0.5 m^–1^ μm depth interval was taken at a resolution of 1,024 × 1,024 pixels. For fluorescence analysis, the confocal settings were constant for all scans. MetaMorph image analysis software (Universal Imaging Corporation, Downingtown, PA) was used to analyze and quantify the whole-cell morphometry. A 20× objective was used to measure the total dendrite length. All dendrites of a single neuron were tracked, and the number of pixels was automatically counted and converted to micrometers with MetaMorph. For the dendrite tip number, the tip of all non-axonal protrusions over 10 μm was calculated. For Sholl analysis, we drew concentric circles with a diameter of 15 μm around the cell body and manually counted the number of dendrites passing through each circle. For dendritic spine analysis, neurons were imaged with a Leica SP8 microscope with a 63× objective and 2 zooms with a 1,024 × 1,024 pixel resolution. At least 36 cultured neurons from three batches of neurons were used for quantitative analysis of each genotype. Quantitative analysis of dendritic spines was performed by using the NeuronStudio software package (Zhu et al., 2016).

### Quantitative Real-Time PCR

TRIzol (Invitrogen, United States) was used to extract the total RNA from cultured neurons following the manufacturer’s instructions. cDNA was synthesized using a cDNA synthesis kit (Thermo Fisher Scientific) according to the standard protocol. RT-qPCR was conducted using Brilliant SYBR Green QPCR Master Mix (Stratagene) on an MX3000P System (Stratagene). The primer sequences were as follows: Cic, forward primer, 5′-ACATCACAGCCTCAGAAGGTCC-3′, reverse primer, 5′-AGAACTGGTGCCTAGAGGCAGA-3′. Etv4, forward 5′-CACAGACTTCGCCTACGACTCA-3′, and reverse, 5′-GCAGACATCATCTGGGAATGGTC-3′. Etv5, forward 5′-GCAGGAATACCATGACCCACTG-3′, and reverse, 5′-AGGATGACTGGCAGTTAGGCAC-3′. GAPDH, forward 5′-GGTGAAGGTCGGTGTGAAC-3′, and reverse, 5′-GAGTG GAGTCATAACTGGAAC-3′. Relative amounts of product transcripts were quantified by the comparative threshold cycle method (ddCt), and GAPDH was used as an endogenous reference control.

### Statistical Analysis

Data are represented as the mean ± SE from at least three biological replicates for experiments. Statistical differences were determined by Student’s *t*-test for two-group comparisons or ANOVA followed by Tukey’s test for multiple comparisons among more than two groups.

## Results

### Capicua Is Highly Expressed in Hippocampal Neuronal Dendrites

**FIGURE 1:**
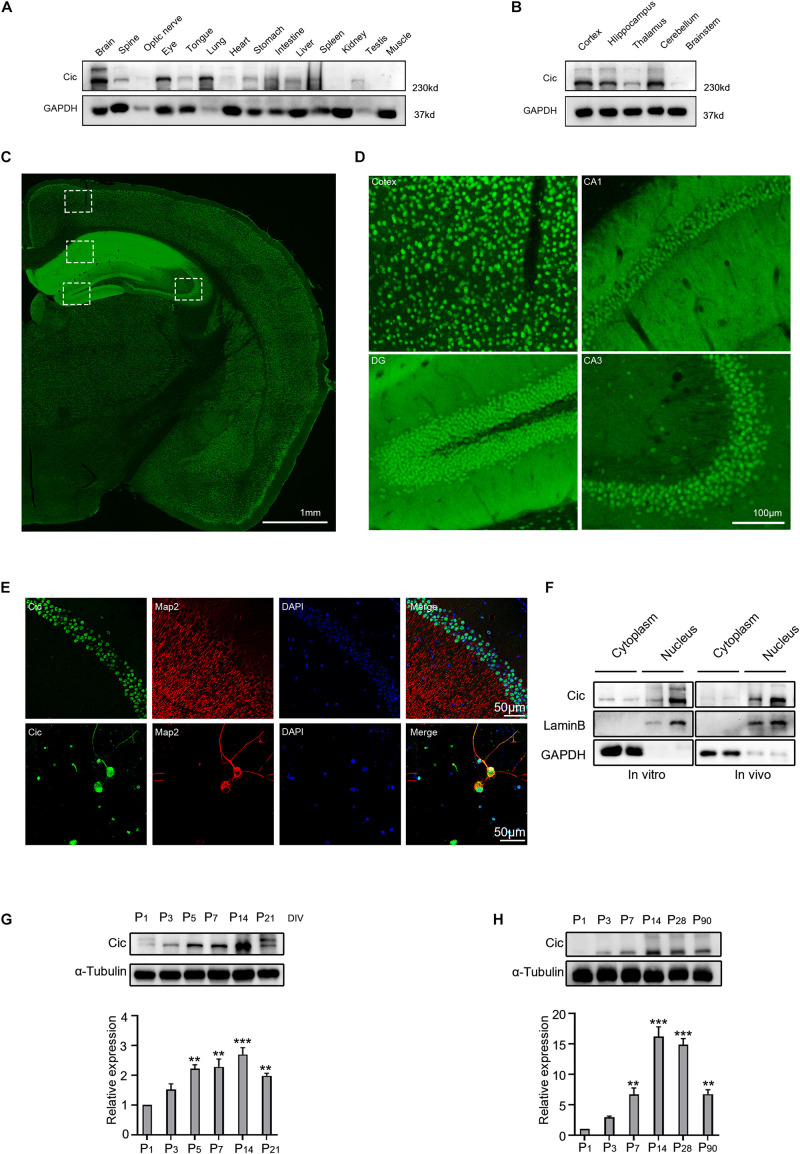
Detection of Cic expression in hippocampal neurons. **(A,B)** The expression of Cic in different tissues and different regions of the brain was analyzed by western blot. **(C)** The expression of Cic in the brain was analyzed by immunofluorescence. **(D)** Higher magnification view of the boxes in **(C)**. **(E)** Expression of Cic (green) with the neuron dendritic marker Map2 (red), as shown by immunofluorescence in brain slices and cultured hippocampal neurons after DIV 14. Nuclear staining with DAPI is also shown. The last panel shows merged images. **(F)** The nuclear and cytoplasmic expression of Cic in cultured hippocampal neurons at DIV 14 (*in vitro*) and in the hippocampus (*in vivo*) was detected by western blot. **(G)** Hippocampal culture lysates from DIV 1 to 21 were immunoblotted to detect Cic, and α-tubulin was used as a loading control. The bottom panel shows the quantification of Cic levels in western blots as in **(G)**, normalized to α-tubulin; *n* = 3 independent hippocampal cultures. **p < 0.01, ***p < 0.001 versus the P1 group. **(H)** Mouse hippocampal homogenates obtained at the indicated stages were immunoblotted to detect Cic, and α-tubulin was used as a loading control. The lower panel shows the quantification of Cic levels in western blots as in **(H)**, normalized to α-tubulin; *n* = 3 independent homogenates. **P < 0.01, ***p < 0.001 versus the P1 group.

To investigate Cic expression in mouse tissues, we first detected the expression of Cic in different tissues of adult mice by western blot. As shown in Figure 1A, Cic was expressed in almost all tissues, especially in the brain, spinal cord, optic nerve, eyes, lung, intestine and spleen (Figure 1A). To explore the distribution of Cic in the brain, we analyzed the expression pattern of Cic by immunoblotting and immunofluorescence using a specific antibody. Cic was highly expressed in different areas of the brain, such as the cortex, hippocampus, thalamus, and cerebellum, but expressed at relatively low levels in the brainstem area (Figure 1B). Prominent labeling of Cic was evident in the cortex, CA1 and CA3 pyramidal neurons, and the granule cell layer of the dentate gyrus (Figures 1C,D). Further studies indicated that Cic was expressed not only in the nucleus but also colabeled with the neuronal marker Map2 in cultured neurons, while *in vivo* Cic was found to be expressed in the nucleus (Figure 1E), indicating that Cic was mainly expressed in neurons. We further extracted cytoplasmic and nuclear proteins to determine the subcellular levels of Cic both *in vitro* and *in vivo*. As shown in Figure 1F, Cic was mainly expressed in the nucleus as demonstrated by both *in vitro* and *in vivo* studies; however, Cic expression was also detected in the cytoplasm. Western blotting was then performed to investigate Cic expression during hippocampal development, and Cic was present in cultured neurons from 0 to 21 DIV, displaying an upregulation at day 5 (Figure 1G), and was present in extracts of the mouse hippocampus throughout development (P1 to P90), with increased expression at P7-90 compared to P1 (Figure 1H).

### Knockdown of Cic Reveals Roles in Dendrite Morphogenesis and Dendritic Spine Growth

**FIGURE 2:**
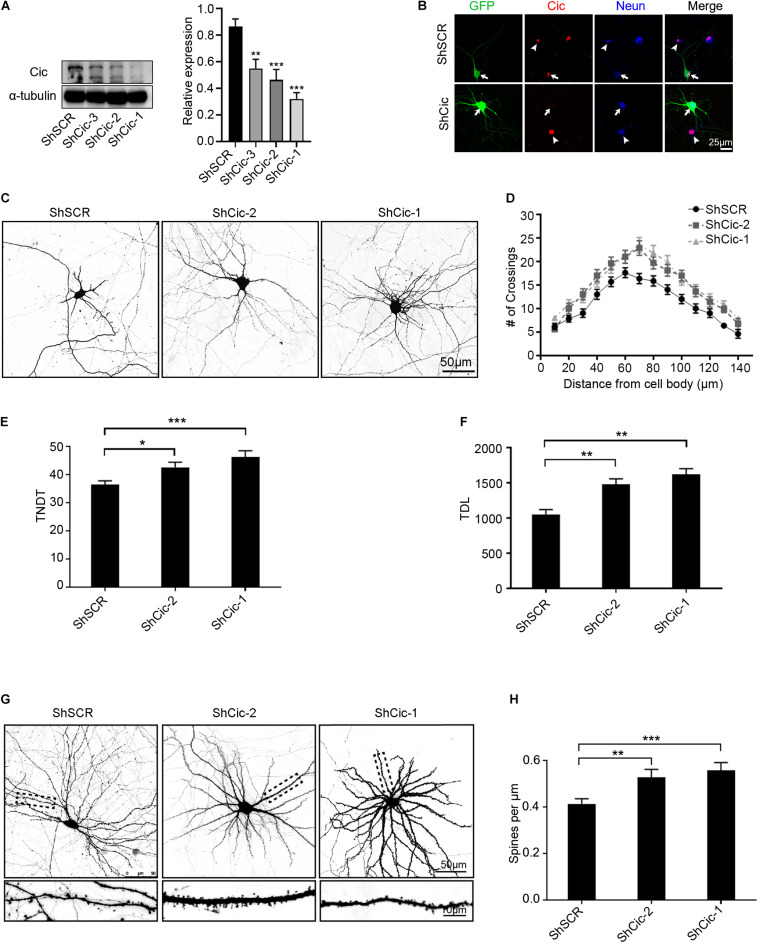
Knockdown of Cic reveals roles in dendrite morphogenesis and the growth of dendritic spines. **(A)** Knockdown of Cic by lenti-shRNA infection of hippocampal neurons cultured *in vitro* from DIV 6–7 for 6–7 days was confirmed by WB. The right panel shows quantification of WB. **(B)** Hippocampal neurons cultured *in vitro* were transfected on DIV 7 for 6 days with either scramble ShSCR-GFP or ShRNA against Cic. Afterward, the cells were stained with an antibody against Cic, the arrow indicates transfected neurons, and the arrowhead indicates non-transfected cells. **(C)** Representative images of hippocampal neurons transfected at DIV6-7 with ShSCR, ShCic-1 or ShCic-2 for 6–7 days. **(D)** Sholl analysis of neurons transfected with ShSCR, ShCic-1 or ShCic-2 (ShSCR: *n* = 52; ShCic-1: *n* = 52; ShCic-2: *n* = 52). **(E,F)** TNDT and TDL of neurons after Cic knockdown (ShSCR: *n* = 52; ShCic-1: *n* = 52; ShCic-2: *n* = 52). **(G)** Representative images of neurons transfected on DIV 7 with ShSCR, ShCic-1 or ShCic-2 for 14 days. **(H)** Quantification of dendritic spine densities (ShSCR: *n* = 52; ShCic-1: *n* = 52; ShCic-2: *n* = 52). Data are shown as the mean ± SE and are representative of *n* = 3 independent experiments. ***p < 0.001; **p < 0.01; *p < 0.05.

To determine the functions of Cic in neurons, we identified small hairpin RNAs (ShRNAs) that allowed the efficient knockdown of Cic as determined by western blot (Figure 2A). To further confirm the effectiveness of the ShRNAs, IF was performed using a specific antibody against Cic. As shown in Figure 2B, ShRNAs directed against Cic effectively decreased Cic protein levels compared with untransfected neurons or scramble-transfected cells. We then transfected shRNA plasmids or scramble plasmids expressing enhanced green fluorescent protein (EGFP) into hippocampal neurons at DIV 7, fixed them at DIV 14, and performed morphometric analysis to measure dendrite morphology. Sholl analysis, which measures the number of dendrites crossing concentric circles at different radial distances from the cell soma, was used to quantify the branching patterns of the dendritic trees. In neurons transfected with scramble plasmid at DIV 14, the number of crossings increased with the distance from the cell soma and reached a maximum at ∼60 μm, while beyond that distance, the number of crossings decreased until 140 μm from the cell body (Figures 2C,D). However, knockdown of Cic increased the complexity of dendritic arborization compared to the scramble control (Figures 2C,D). Among the neurons transfected with ShRNA plasmids, the number of crossings reached a peak at approximately 70 μm from the cell body, and the number of crossings at 140 μm was higher in the shRNA-transfected cells than in the scramble-transfected cells (Figure 2D). Knockdown of Cic also led to a strong increase in the total number of dendritic tips (TNDT; 16 and 27%, respectively, Figures 2C,E) and the total dendrite length (TDL; 41 and 54%, respectively, Figures 2C,F) compared to the control condition. In addition to the significant changes in overall dendritic morphology mentioned above, Cic knockdown also led to significant changes in dendritic spine density. As shown in Figures 2G,H, 14 days after transfection at 7th day, Cic knockdown increased the spine density (by 42 and 49% for ShCic-1 and ShCic-2, respectively, compared with ShSCR). Taken together, these results suggest that a major role of Cic in neurons is the regulation of dendrite growth.

### Overexpression of Cic in Hippocampal Neurons Suppresses Dendrite and Dendritic Spine Growth *in vitro*

**FIGURE 3:**
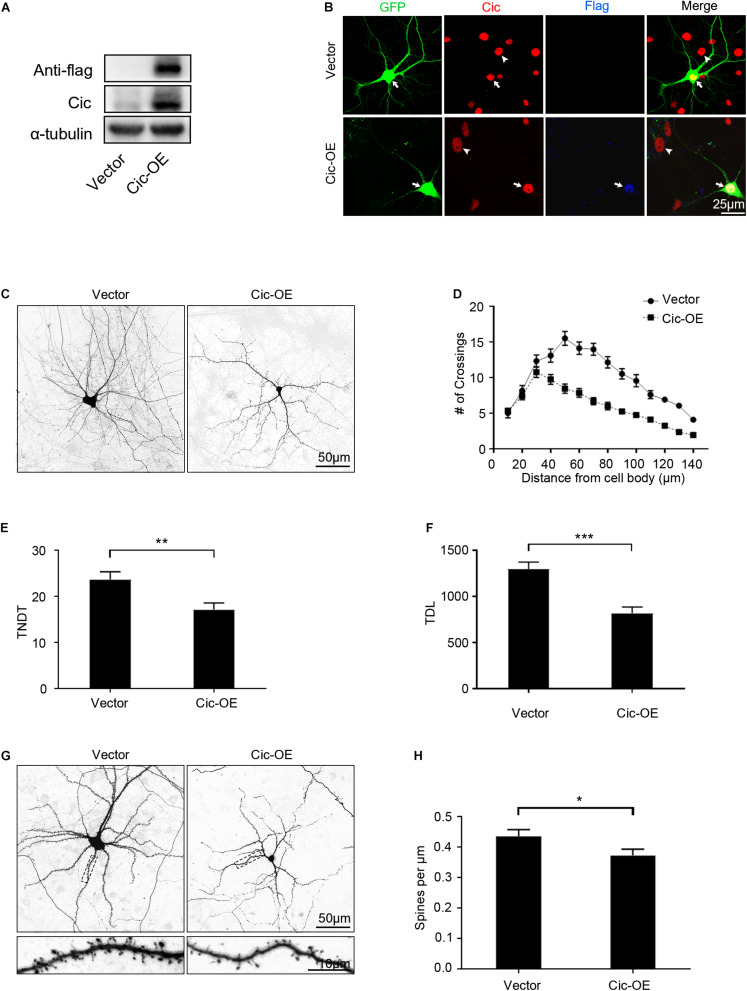
Overexpression of Cic in hippocampal neurons suppresses dendrite and dendritic spine growth *in vitro*. **(A)** The Cic protein expression level in HT22 cells after Cic-OE or empty vector transfection was detected by western blot. **(B)** Hippocampal neurons cultured *in vitro* were transfected on DIV 7 for 6 days with either control vector or Cic-OE-GFP. Afterward, the cells were stained with antibodies against Cic and Flag, the arrow indicates transfected neurons, and the arrowhead indicates untransfected cells. **(C)** Representative images of deisolated neurons transfected on DIV 7 for 7 days with vector-GFP or Cic-OE-GFP. **(D–F)** Sholl analysis, TNDT and TDL of deisolated neurons transfected with vector or Cic (vector: *n* = 50; Cic-OE: *n* = 50). **(G)** Representative images of deisolated neurons transfected on DIV 7 for 14 days with vector or Cic-OE. **(H)** Quantification of dendritic spine densities (vector: *n* = 36; Cic-OE: *n* = 36). Cell images were obtained from three independent culture batches. Error bars indicate SE. ***p < 0.001; **p < 0.01; *p < 0.05.

The finding that Cic knockdown in hippocampal neurons suppresses the growth of dendrites prompted us to speculate that Cic plays a negative role in dendrite growth in hippocampal neurons *in vitro*. Thus, to confirm this hypothesis, we overexpressed Cic in dissociated cultured hippocampal neurons. First, immunoblotting was performed to verify Cic overexpression in HT22 cells (Figure 3A). IF was then performed, and as shown in Figure 3B, Cic staining was increased compared with that in the untransfected cells or in the vector-transfected control, while Flag staining was only present in Cic-OE-transfected cells.

Contrary to the effect of Cic knockdown in neurons, overexpression of Cic in cultured neurons inhibited dendrite branching, TNDT and TDL (Figure 3C). Sholl analysis showed that the “peak” of dendritic branching in hippocampal neurons transfected with Cic-OE was shifted leftward and downward (Figures 3C,D). We also found that Cic overexpression significantly decreased the TNDT and TDL of neurons. The Cic-OE neurons showed an ∼27% decrease in TNDT and an ∼37% decrease in TDL compared with the vector-neurons (Figures 3C,E,F). Cic overexpression also suppressed the growth of dendritic spines. Fourteen days after transfection with plasmids Cic-OE on the 7th day, the neurons showed an ∼14% decrease in the density of spines (Figures 3G,H).

### Cic Represses Ets Factor Expression in the Dendritic Growth of Hippocampal Neurons

**FIGURE 4:**
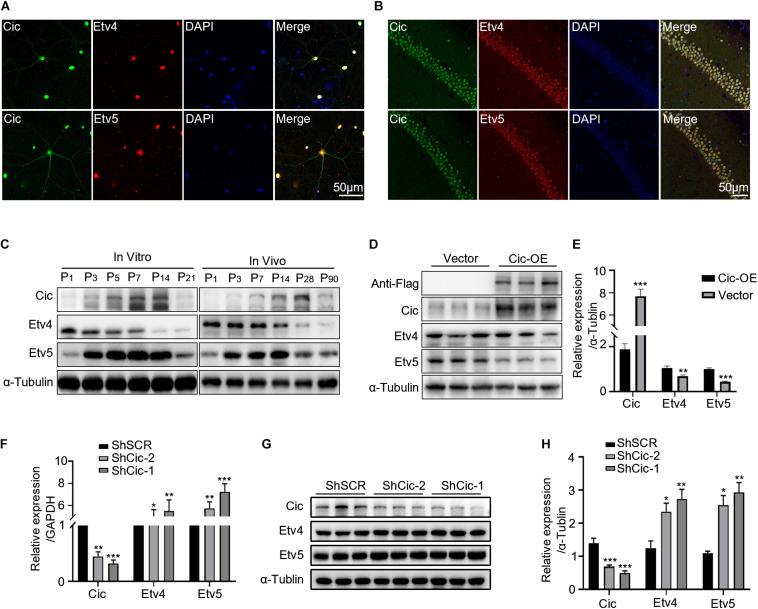
Cic represses Ets factor expression in the dendritic growth of hippocampal neurons. **(A)** Localization of Cic (green) and Etv4 (top) (red) or Etv5 (bottom) (red) in dissociated mouse hippocampal neurons after DIV13. Nuclear staining with DAPI is also shown. Last panel showed merged images. **(B)** Localization of Cic (green) and Etv4 (top) (red) or Etv5 (bottom) (red) in coronal sections of the mouse hippocampus. **(C)** Hippocampal culture lysates from DIV 1 to 21 and mouse hippocampal homogenates obtained at the indicated stages were immunoblotted to detect Cic, Etv4, and Etv5. α-Tubulin was used as a loading control. **(D)** HT22 cells were infected for 3 days with either lenti-control vector or lenti-Cic-OE, and WB was used to ascertain the change in Cic and Ets following overexpression of Cic in HT22 cells. **(E)** shows quantification of WB in d. **(F–H)** Hippocampal neurons cultured *in vitro* were infected on DIV 7 for 7 days with either lenti-scramble ShSCR or lenti-ShRNA against Cic (ShCic-1, ShCic-2). **(F)** mRNA levels of Ets were assessed by qPCR following Cic knockdown in hippocampal neurons. **(G)** The expression of Ets following Cic knockdown in neurons was detected by WB. **(H)** Quantification of the WB in G. The error bars indicate the SE. ***p < 0.001; **p < 0.01; *p < 0.05.

The Pea3 transcription factors Etv4 and Etv5 have been identified as Cic targets (Ahmad et al., 2019; Hwang et al., 2020). Thus, we decided to examine whether these genes were regulated by Cic in the dendritic growth of hippocampal neurons. First, we found that Cic colocalized with Etv4 and Etv5 both in hippocampal neurons *in vitro* and *in vivo* by IF (Figures 4A,B). Then, the expression of Cic and Ets in the developing mouse hippocampus was determined by the western blot. We found that the expression of Cic and Etv5 gradually increased in cultured neurons from 0 to 14 DIV and during the first 2 weeks of postnatal development *in vivo*, while Etv4 expression gradually decreased both *in vitro* and *in vivo* (Figure 4C). These studies further indicated that Ets may be a target for Cic in hippocampal neurons. Indeed, Cic overexpression in HT22 cells reduced the protein levels of Etv4 and Etv5 (Figure 4D), while Cic knockdown in hippocampal cultures significantly increased Etv4 and Etv5 mRNA (Figure 4F) and protein levels (Figures 4G,H). Taken together, these results raise the possibility that Cic may regulate dendrite growth through Ets.

### Dendritic Growth Promoted by Cic Silencing Was Suppressed by Knockdown of Ets

**FIGURE 5:**
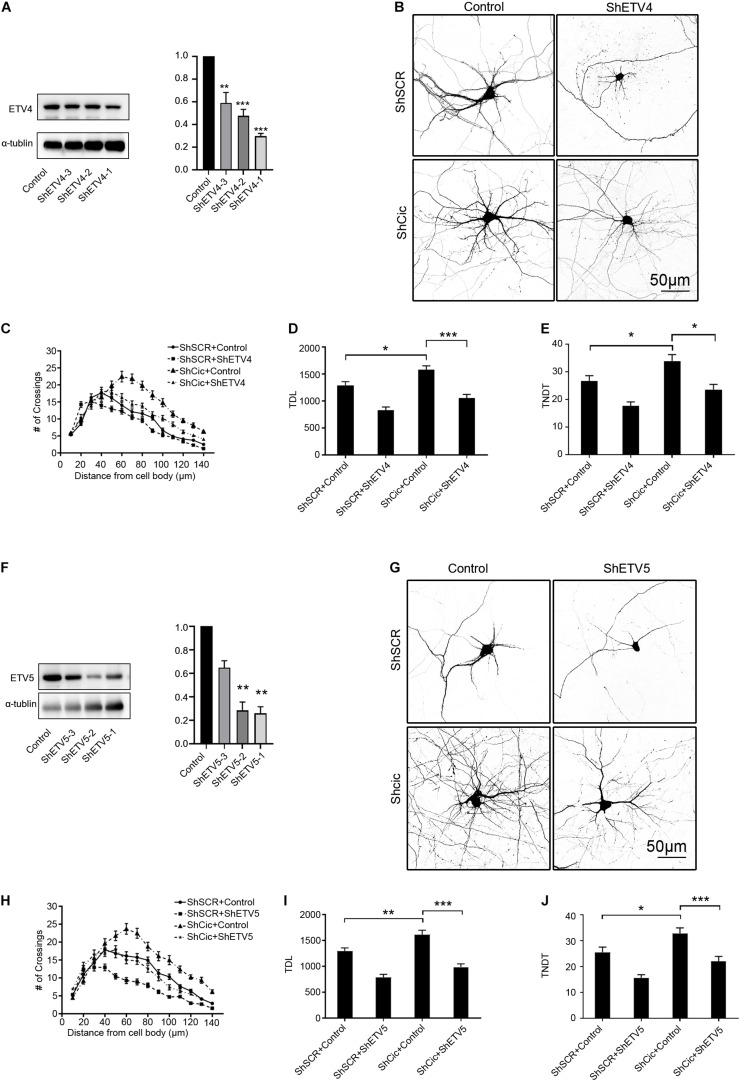
Cic silencing induced promotion of dendritic spine growth was suppressed by Ets knockdown. **(A)** The Etv4 protein expression level in HT22 cells after ShEtv4 or scramble plasmid transfection was detected by western blot. The right panel shows the quantification of the WB. **(B)** Hippocampal neurons cultured *in vitro* were cotransfected on DIV 7 for 7 days with control or ShEtv4 and scramble ShSCR or ShCic. **(C–E)** Neuronal morphology was visualized by cotransfection with enhanced green fluorescent protein. Sholl analysis (ShSCR/Control, *n* = 48; ShSCR/ShEtv4, *n* = 48; ShCic/Control, *n* = 48; ShCic/ShEtv4, *n* = 48) **(C)**, TDL (*n* as in **C**) **(D)**, and TNDT (*n* as in **C**) **(E)** of neurons after transfection with the indicated plasmids. **(F)** The Etv5 protein expression level in HT22 cells after ShEtv5 or empty vector transfection was detected by western blot. The right panel shows the quantification of the WB. **(G–J)** Representative images of hippocampal neurons transfected on DIV 7 for 7 days with control or ShEtv5 and scramble ShSCR or ShCic. Neuronal morphology was visualized by cotransfection of enhanced green fluorescent protein. Sholl analysis (ShSCR/Control, *n* = 48; ShSCR/ShEtv5, *n* = 48; ShCic/Control, *n* = 48; ShCic/ShEtv5, *n* = 48) **(H)**, TDL (*n* as in **H**) **(I)**, and TNDT (*n* as in **H**) **(J)** of neurons after transfection with the indicated plasmids. The error bars indicate the SE. ***p < 0.001; **p < 0.01; *p < 0.05.

To determine whether Ets plays a role in Cic-induced inhibition of dendrite morphogenesis, ShRNA was used to knock down Ets in Cic-knockdown neurons. First, HT22 cells were transiently transfected with ShRNA or control plasmids. All ShRNAs targeting Ets had a high knockdown efficiency at the protein level (Figures 5A,F). Then, neurons were cotransfected with ShCic and either ShEtvs or a suitable ShRNA vector. Neurons cotransfected with ShCic and vector served as an additional control. In addition, all groups were cotransfected with plasmids encoding GFP to visualize the morphology of the transfected neurons. Knockdown of Etv4 and Etv5 resulted in a significant decrease in the complexity of hippocampal dendritic arborization, TDL and TNDT compared with ShSCR/control transfection (Figures 5B,D,E,G,I,J). Furthermore, simultaneous knockdown of Cic and Etv4 or Etv5 significantly prevented the phenotype of Cic knockdown. Sholl analysis showed that compared with those in ShCic/control-transfected cells, the maximum number of crossings was decreased and the “peak” of branching was shifted leftward in ShCic/ShEtv4- or ShCic/ShEtv5-transfected cells (Figures 5B,C,G,H). These observations indicate that Cic negatively regulates Etv4 and Etv5 expression, which is important for the regulation of hippocampal dendritic arbors by Cic.

## Discussion

Stereotypic dendrite arborization and spines are the key morphological features of neurons and are essential for integrating neuronal information. Precise dendrite patterns and their coordination with synaptic activity are necessary to ensure proper neuronal function and connectivity (Ledda and Paratcha, 2017). Numerous studies in the past have shown that transcription factors are major players in controlling multiple aspects of neuronal morphogenesis, including dendritic growth and branching and synapse formation (de la Torre-Ubieta and Bonni, 2011). Accumulating evidence has shown that an interesting feature of dendritic growth regulation is that transcription factors such as MeCP2 (Zhou et al., 2006; Cheng et al., 2014), FOXO6 (de la Torre-Ubieta et al., 2010), and Sp4 (Ramos et al., 2007) act as negative regulators of dendritic growth. Thus, studies of transcriptional repressors provide a basis for elucidating the key mechanisms of dendritic morphogenesis, which may also provide a better understanding of the molecular basis of brain development disorders.

Cic, an evolutionarily conserved transcription factor from *Caenorhabditis elegans* to humans (Jimenez et al., 2000; Fores et al., 2017), is substantially downregulated in prostatic carcinoma (PC) (Choi et al., 2015), hepatocellular carcinoma (HCC) (Kim et al., 2018) and colorectal cancer (CRC) (Lee et al., 2020), suggesting that Cic may likely act as a tumor suppressor in cancer. In brain tumors, Cic mutations were found in oligodendroglioma (Bettegowda et al., 2011; Yip et al., 2012), oligoastrocytoma (Sahm et al., 2012), astrocytoma (Sahm et al., 2012), glioblastoma (Bunda et al., 2019) and medulloblastoma (Lee et al., 2005). The identification of the Cic cancer gene provides an opportunity to reveal previously unknown mechanisms that regulate brain development. Indeed, previous reports found that a genetic deficiency of Cic in the brains of mice increases oligodendrocyte progenitor cell (OPC) and immature oligodendrocyte populations, likely at the expense of neuronal differentiation (Yang et al., 2017; Ahmad et al., 2019). In contrast, another study found that brain-specific Cic impairs the developmental transition of neuroblasts to immature neurons in the hippocampus of mice and impairs normal neuronal differentiation (Hwang et al., 2020). Regarding the function of Cic in a non-neoplastic context, Lu et al. (2017) showed that disruption of the interaction of Cic with Ataxin1 disrupted the organization and maintenance of neurons in the upper cortex, leading to hyperactivity and impaired learning and memory in mice, which suggests a role for Cic in neuronal biology.

In this study, we found that Cic is highly expressed in the central nervous system, especially in the cortex and hippocampus, which is consistent with previous reports of Cic expression in the brain (Lee et al., 2011; Kim et al., 2015; Ahmad et al., 2019; Hwang et al., 2020). We also found that the expression of Cic in hippocampal neurons reaches the highest level in the early postnatal period, that is, at the time when dendrites and synapses develop (Ben-Ari et al., 2007), indicating that Cic may have a function in dendrite growth in hippocampal neurons. Indeed, we found that knocking down Cic by using ShRNAs promoted the branching of dendrites and the growth of dendritic spines (Figure 2), while Cic overexpression inhibited the branching of dendrites and the growth of dendritic spines (Figure 3). However, Lu et al. (2017) showed that mouse forebrain–specific deletion of Cic using an Emx1-Cre allele caused defective dendritic branching in layer 2/3 pyramidal neurons, while no difference in dendritic branching complexity was observed in layer 5 pyramidal neurons between mutant and control animals, which indicates that the function of Cic may be inconsistent in different brain regions or different neurons. The difference between the results in this study and in the reports mentioned above may be caused by the following reasons: (1) the role of Cic may be different in the cortex and hippocampus; (2) the growth conditions for neurons are different *in vivo* and *in vitro*; and (3) the mechanism compensating for the loss of Cic is different. Our studies focused on changes in the dendritic arbor morphology of hippocampal neurons, while the increased number of dendrites may result from either the increased formation of new branches or the inhibition or retraction of existing branches, as rapid extension and retraction of dendrites occurs frequently during the growth of dendrites (Williams and Truman, 2004); thus, future work using time-lapse microscopy will be needed to define the function of Cic in dendrites.

As Cic is an evolutionarily conserved transcription repressor (Lee, 2020), a possible mechanism for our findings is that Cic depresses specific genes that drive dendrite growth in neurons. Thus, Etv4 and Etv5 are candidate targets of interest. Etv4 and Etv5 are expressed in various organs and tissues during embryonic development and in adults, as shown by mRNA distribution analyses (Lu et al., 2009; Zhang et al., 2009). In the brain, Etv4 and Etv5 are critical mediators of retrograde nerve growth factor signaling, gene expression, and axonal growth of DRG sensory neurons (Fontanet et al., 2013). Moreover, Etv4 and Etv5 are also involved in the axonal growth of DRG neurons expressing the BDNF receptor TrkB (Liu et al., 2016). In terms of neuronal dendrites, Etv4 and Etv5 were found to be key components of the downstream gene network of BDNF/TrkB, which promotes and controls hippocampal dendritic morphology (Fontanet et al., 2018). These observations led us to examine whether Cic regulates dendrite growth through Etv4 and Etv5. Indeed, we found that Cic knockdown increased Etv4 and Etv5 expression at the mRNA and protein levels (Figure 4). Given the known role of Etv4 and Etv5 in promoting dendritic outgrowth (Fontanet et al., 2018), persistent Etv4 and Etv5 activation due to Cic knockdown is a plausible mechanism for dendritic growth in this study.

## Conclusion

In conclusion, this study demonstrates that the transcriptional repressor Cic is expressed in the brain and hippocampal pyramidal neurons. Our experiments indicate that Cic negatively regulates hippocampal dendrite growth possibly through Etv4 and Etv5. It will be important to analyze whether Cic has implications for human brain disorders characterized by cognitive alterations.

## Data Availability Statement

The original contributions presented in the study are included in the article/supplementary material, further inquiries can be directed to the corresponding author/s.

## Ethics Statement

The animal study was reviewed and approved by the Animal Welfare Committees of Tongji University in Shanghai.

## Author Contributions

CZ, HL, KC, and JZ: conceptualization and writing—review and editing. KL, KC, SS, TJ, and LWa: methodology. ML, YP, MC, SX, LWe, KZ, QW, YW, and YZ: formal analysis. KL, KC, and SS: writing—original draft preparation. CZ: supervision, project administration, and funding acquisition. All authors contributed to the article and approved the submitted version.

## Conflict of Interest

The authors declare that the research was conducted in the absence of any commercial or financial relationships that could be construed as a potential conflict of interest.

## Publisher’s Note

All claims expressed in this article are solely those of the authors and do not necessarily represent those of their affiliated organizations, or those of the publisher, the editors and the reviewers. Any product that may be evaluated in this article, or claim that may be made by its manufacturer, is not guaranteed or endorsed by the publisher.
